# Associations of sleep disorders with all-cause and cause-specific mortality in cancer survivors: a cross-sectional analysis of the NHANES 2005–2016

**DOI:** 10.1186/s12888-024-05589-3

**Published:** 2024-02-12

**Authors:** Jingying Nong, Jinzhuo Tong, Ruotian Wang, Kejian Shi, Yi Zhang

**Affiliations:** 1 Department of Thoracic Surgery, Xuanwu Hospital, Capital Medical University, Changchun Street 45#, Beijing, 100053 China; 2 Experimental School of Beihang University, Xueyuan Road 37#, Beijing, 100083 China

**Keywords:** Sleep, Cancer, Mortality, Cardiovascular disease, Cancer mortality

## Abstract

**Background:**

The circadian clock and endoplasmic reticulum stress signaling play important roles in oncogenesis and development of cancer. Sleep disorders have been linked to an elevated risk of mortality in general populations. Nonetheless, the evidence for the sleep disorders-mortality association among cancer patients is limited. We aimed to prospectively investigate the association of sleep disorders with all-cause, cancer, and cardiovascular disease (CVD) mortality among cancer individuals.

**Methods:**

We assessed 3187 participants with cancer from the National Health and Nutrition Examination Survey 2005–2016 cohorts with a median follow-up time of 83.0 months. Multivariable Cox proportional hazards models estimated the adjusted hazard ratio (HR) and 95% confidence interval (CI).

**Results:**

Multivariable Cox proportional hazards models showed that sleep disorders were associated with a higher risk of all-cause mortality (HR 1.23, 95%CI: 1.06,1.42), cancer mortality (HR 1.30, 95%CI: 1.02, 1.66), and cardiovascular disease mortality (HR 1.35, 95%CI: 1.02, 1.80). After the total group was stratified by gender, the high HRs were observed in men (*P* < 0.05), not in women. The correlation between sleep disorders and higher long-term mortality was also significant after individuals who died within 2 years of follow-up were excluded, with HR 1.24 (95%CI: 1.07, 1.45) in model I, HR 1.20 (95%CI: 1.02, 1.42) in model II for long-term all-cause mortality, HR (95%CI: 1.00, 1.74) in model I for long-term cancer mortality, and HR 1.5 (95%CI:1.12, 2.02) in model I, HR 1.45 (95%CI: 1.06, 1.99) in model II for long-term CVD mortality.

**Conclusions:**

Sleep disorders were associated with a higher risk of all-cause mortality, cancer mortality, and CVD mortality, as well as long-term mortality in cancer patients. Our finding underlies the importance of screening for sleep disorders for all cancer survivors and the urge to integrate sleep health as an important part of cancer care more effectively. Male individuals may be particularly vulnerable and could benefit from more frequent screening.

**Supplementary Information:**

The online version contains supplementary material available at 10.1186/s12888-024-05589-3.

## Introduction

Sleep is crucial to all human functioning. Humans spend about one-third of their time sleeping, which includes a complex physiological and behavioral course. Disrupting these processes can result in different types of sleep disorders singly or in combination. Sleep disorders [1] are frequent in the general population and currently have been recognized as a public health disease by the Centers for Disease Control [2].

Sleep disorders include insomnia, sleep-related breathing disorders, central disorders of hypersomnolence, circadian rhythm sleep-wake disorders, parasomnias, sleep-related movement disorders, and other sleep disorders [1]. In general populations, sleep disorders have been significantly linked to reduced quality of life and elevated risks of metabolic disease, arterial stiffness [3], endothelial dysfunction [4], cardiovascular events [5–7], and increased risk of mortality [6, 8–10].

Cancer is a major public health problem worldwide and one of the leading causes of death in the United States, with 599,274 in 2018 according to the National Center for Health Statistics, Centers for Disease Control and Prevention [11]. Over the last decade, with considerable progression in the diagnosis and treatment of cancer, the number of cancer survivors has been estimated to grow as the population ages, many of them could either expect long-term survival or live with malignancies as chronic diseases controlled by continuing therapy. Despite the achievements in cancer early detection and therapy, long-term behavioral comorbidities are prominent. Among these, Sleep disorders are prevalent among cancer survivors, with a significantly higher incidence than in the general population [12].

The circadian clock and endoplasmic reticulum stress signaling play important roles in oncogenesis and development of cancer [13]. Although the association of sleep disorders with cancer risk has been reported in a few studies [14], evidence indicating the relationship between sleep disorders and mortality among cancer individuals is limited [15, 16], and lack of information on the actual cause of death. In addition, since a bidirectional connection between sleep and cancer exists, few studies considered the influence of the timing on questionnaires of sleep disorders to the follow-up time, there is a greater gap in knowledge about the effect of sleep disorders on long-term cancer outcomes.

This study aimed to prospectively investigate the relationship between sleep disorders and all-cause, cancer, and cardiovascular disease (CVD) mortality among participants with cancer, and whether the associations were influenced by follow-up time in a nationally representative US sample utilizing data from 6 cycles of the National Health and Nutrition Examination Survey (NHANES) (2005–2016) linked with the most updated 2019 National Death Index (NDI).

## Methods

### Study population

**Fig. 1:**
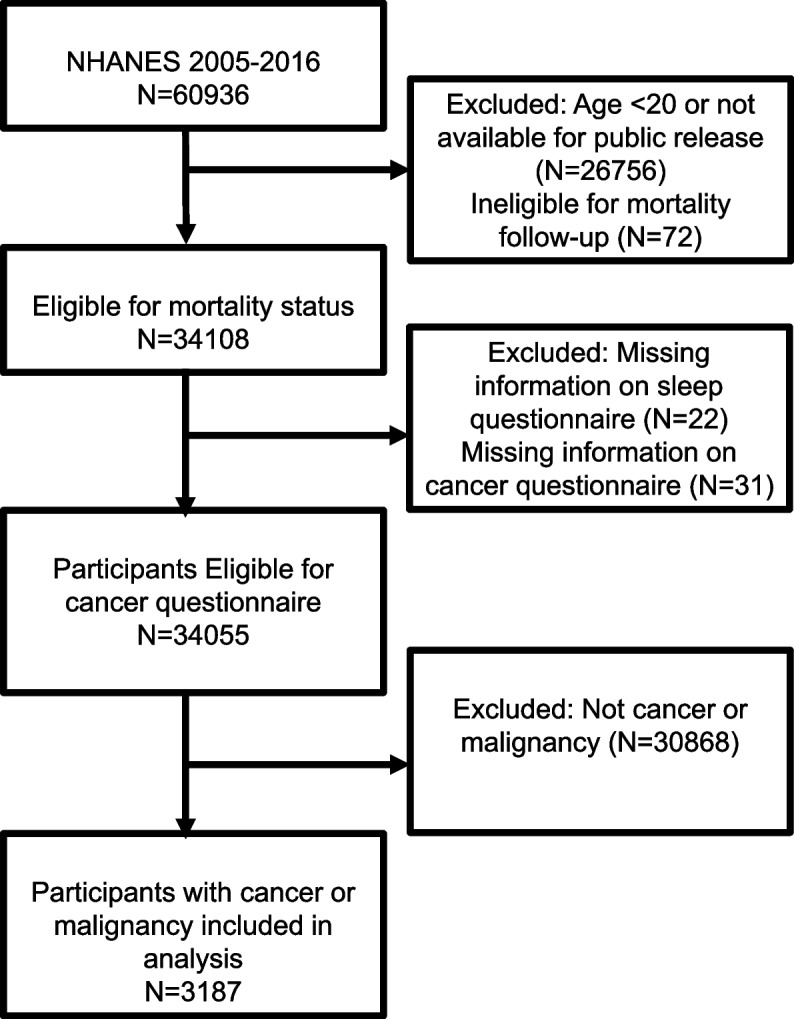
The flow chart of the study. NHANES, National Health and Nutrition Examination Survey

This study was performed using the NHANES dataset with a complex, multi-stage, stratified, clustered probability design [17], which has been described online (https://www.cdc.gov/nchs/index.htm). The NHANES study protocol was approved by the National Center for Health Statistics Institutional Review Board and informed written consent was provided by all participants at enrollment. The data were from 6 cycles of the NHANES (2005–2006, 2007–2008, 2009–2010, 2011–2012, 2013–2014, 2015–2016). A total of 60,936 individuals participated in six consecutive NHANES 2-year cycles (2005–2016). The individuals were followed up for mortality status until December 31, 2019. Participants eligible for mortality status assessment were first enrolled, individuals age < 20, not available for public release, or ineligible for mortality follow-up were excluded. After excluding individuals without information on the sleep questionnaire or the cancer or malignancy questionnaire, a total of 34,055 participants left. Then, we excluded those denied cancer or malignancy according to the cancer questionnaire. In total, 3187 participants with cancer were eligible in the final analysis (Fig. 1).

### Cancer status

In the NHANES, the medical conditions section provides self-reported health condition information. Cancer diagnoses were based on the following question: “Have you ever been told by a doctor or other health professional that you had cancer or a malignancy (ma-lig-nan-see) of any kind?” Questions were asked in the home by trained interviewers using the Computer-Assisted Personal Interview (CAPI) system [18]. The CAPI system is programmed with built-in consistency checks to reduce data entry errors.

### Sleep disorders

Sleep disorders were assessed with the Sleep Disorder Questionnaire and participants were asked SLQ050 “Have you ever told a doctor or other health professional that you have trouble sleeping” [19, 20]. And then participants were categorized according to the answers (yes or no), participants with the answers “Do not know” and “Refused” were excluded.

### Mortality

The public-use LMF provides mortality follow-up data from the NHANES Mobile Examination Center (MEC) date through December 31, 2019. The method can be found in the National Center for Health Statistics. The files include mortality status and underlying causes of death. Causes of death were coded according to the 10th International Classification of Diseases (ICD-10). In this study, persons who did not have sufficient identifying data or were not available for public release were excluded.

### Covariates

The household interview collected demographic information and lifestyle factors, including age, gender, race/ethnicity, education level, and smoking history. Race/ethnicity was divided into Mexican American, other Hispanic, non-Hispanic White, non-Hispanic Black, and other races. Those who have never smoked or who have only smoked in their lives less than 100 cigarettes were classified as non-smokers. At the Mobile Examination Center, alcohol intake and body mass index (BMI) were recorded. The consumption of alcohol was defined as having at least 12 alcoholic drinks per year. BMI was calculated by dividing weight (kg) by height in square meters. The following question from the medical conditions section was used to diagnose coronary heart disease among participants: “Has a doctor or other health professional ever told you that you had coronary heart disease?” Smoking history, Alcohol drinking history, and coronary heart disease were recorded by “Yes/No”.

### Statistical analysis

For continuous variables, mean and standard deviation (SD) were presented, while counts and proportions were presented for categorical variables [21]. The comparison in the sleep disorder status subgroups was performed using logistic regression for continuous variables (mean and SD) and the Chi-square test for categorical variables. Multivariable Cox proportional hazards models were conducted to investigate the relationship between sleep disorders and all-cause mortality, cancer mortality, and CVD mortality from the MEC date to either the date of death or censoring (December 31, 2019), whichever came first. Model I was a minimally adjusted model adjusted for age and gender (only in the total group). Model II was adjusted for age, gender (only in total group), race/ethnicity, education level, BMI, smoking history, alcohol drinking history, and coronary heart disease. Gender-stratified subgroup analysis was performed to clarify the impact of sleep disorders. Further stratified Cox regression analysis was conducted to identify variables that modify the association between sleep disorder status and mortalities in the total group and male participants. The likelihood ratio test was used to calculate the *P* value for interactions. The associations with sleep disorders and Cancer and CVD mortality were further estimated after multiple adjustments by competing risks models using the R package "mstate", where non-cancer or non-CVD deaths were modeled as different events [22]. All analyses were performed utilizing R software version 4.1 (http://www.R-project.org; The R Foundation) and EmpowerStats (http://www.empowerstats.com, X&Y Solutions, Inc.). *P* < 0.05 (two-sided) was set for a significant difference.

## Results

### Study participants and baseline characteristics

A total of 60,936 individuals participated in six consecutive NHANES 2-year cycles (2005–2016). The participants were followed up for mortality status until December 31, 2019. After removing participants without cancer, a total of 3187 participants with cancer were included for mortality analysis. Table 1 shows the baseline characteristics of the subjects stratified by sleeping disorder status. The mean (SD) age of participants was 65.62 (14.28) years, 1499 (47.0%) were male, and 2175 (68.3%) were non-Hispanic white individuals. Younger participants, women, individuals with higher education levels, smokers, alcohol drinkers, and higher BMI were more likely to suffer from sleep disorders.

**Table 1:** Baseline characteristics of participants stratified by sleep disorders status

Characteristics	Total	Sleep disorders		*P*-value
	No (*n* = 3187)	No (*n* = 2065)	Yes (*n* = 1122)	
**Age, mean ± sd, y**	65.62 ± 14.28	66.78 ± 14.10	63.48 ± 14.38	< 0.001
**Gender**				< 0.001
Male	1499 (47.03)	1034 (50.07)	465 (41.44)	
Female	1688 (52.97)	1031 (49.93)	657 (58.56)	
**Race/ethnicity**				0.166
Mexican American	210 (6.59)	146 (7.07)	64 (5.70)	
Other Hispanic	195 (6.12)	135 (6.54)	60 (5.35)	
Non-Hispanic white	2175 (68.25)	1380 (66.83)	795 (70.86)	
Non-Hispanic black	458 (14.37)	307 (14.87)	151 (13.46)	
Other race - including multi-racial	149 (4.68)	97 (4.70)	52 (4.63)	
**Education**				0.001
Less than 9th grade	329 (10.33)	219 (10.62)	110 (9.80)	
9-11th grade	414 (13.00)	271 (13.14)	143 (12.75)	
High school graduate	694 (21.80)	446 (21.63)	248 (22.10)	
Some college or aa degree	919 (28.86)	551 (26.72)	368 (32.80)	
College graduate or above	828 (26.01)	575 (27.89)	253 (22.55)	
**Smoking history**				< 0.001
Smoker	1756 (55.15)	1054 (51.12)	702 (62.57)	
Non-smoker	1428 (44.85)	1008 (48.88)	420 (37.43)	
**Alcoholic drinking**				0.004
Yes	1976 (69.58)	1235 (67.75)	741 (72.86)	
No	864 (30.42)	588 (32.25)	276 (27.14)	
**BMI (kg/m**^**2**^**)**	28.96 ± 6.49	28.51 ± 6.00	29.76 ± 7.24	< 0.001
**Coronary heart disease**				0.081
Yes	297 (9.38)	179 (8.71)	118 (10.60)	
No	2871 (90.62)	1876 (91.29)	995 (89.40)	
**Decease within 2 years follow-up**				0.215
Yes	204(6.40)	124(6.00)	80(7.13)	
No	2983(93.60)	1941(93.40)	1042(92.87)	

*BMI* body mass index

### Sleep disorders and mortality outcomes in cancer participants

We calculated the follow-up time using person months between the NHANES MEC date and the date of death or the end of the mortality period. The follow-up with a median time of 83.0 (interquartile range: 51.0–122.0) months documented 1028 deaths, including 347 cancer deaths and 269 CVD deaths (Table 2).

**Table 2:** The relationship between sleep disorders and mortality among cancer participants, NHANES(2005–2016)

Outcomes	Non-adjusted Model		Model I		Model II	
	HR (95% CI)	*P*-value	HR (95% CI)	*P*-value	HR (95% CI)	*P*-value
**Sleep disorders**						
**All-cause mortality No. of deaths/participants (1028/3187)**						
**Total**						
No	1(reference)		1(reference)		1(reference)	
Yes	0.97(0.85, 1.11)	0.66	1.27(1.11, 1.45)	< 0.001	1.23 (1.06, 1.42)	< 0.01
**Male**						
No	1(reference)		1(reference)		1(reference)	
Yes	1.28 (1.07, 1.52)	< 0.01	1.52 (1.27, 1.81)	< 0.001	1.47 (1.22, 1.77)	< 0.001
**Female**						
No	1(reference)		1(reference)		1(reference)	
Yes	0.80(0.65, 0.99)	0.04	1.01 (0.82, 1.24)	0.96	1.00(0.80, 1.25)	1.00
**Cancer mortality No. of deaths/ participants (347/3187)**						
**Total**						
No	1(reference)		1(reference)		1(reference)	
Yes	1.09 (0.87, 1.37)	0.45	1.33 (1.06, 1.67)	< 0.05	1.30 (1.02, 1.66)	< 0.05
**Male**						
No	1(reference)		1(reference)		1(reference)	
Yes	1.72 (1.30, 2.28)	< 0.001	1.88 (1.42, 2.49)	< 0.001	1.82 (1.34, 2.46)	< 0.001
**Female**						
No	1(reference)		1(reference)		1(reference)	
Yes	0.64 (0.43, 0.94)	0.02	0.73(0.49, 1.09)	0.12	0.74 (0.48, 1.14)	0.17
**CVD mortality No. of deaths/ participants (269/3187)**						
**Total**						
No	1(reference)		1(reference)		1(reference)	
Yes	1.02(0.78, 1.33)	0.88	1.47 (1.13, 1.92)	< 0.01	1.35 (1.02, 1.80)	< 0.05
**Male**						
No	1(reference)		1(reference)		1(reference)	
Yes	1.11 (0.78, 1.58)	0.56	1.49 (1.05, 2.13)	< 0.05	1.49 (1.03, 2.16)	< 0.05
**Female**						
No	1(reference)		1(reference)		1(reference)	
Yes	1.10 (0.74, 1.65)	0.63	1.45 (0.97, 2.18)	0.07	1.22 (0.79, 1.89)	0.37

Model I: adjusted for age, gender (only for overall)

Model II: adjusted for age, gender (only for overall), education levels, race, smoking history, alcoholic drinking, BMI, coronary heart disease

NHANES, National Health and Nutrition Examination Survey; BMI, body mass index; CVD, cardiovascular disease; HR, hazard ratio; CI, confidence interval

**Fig. 2:**
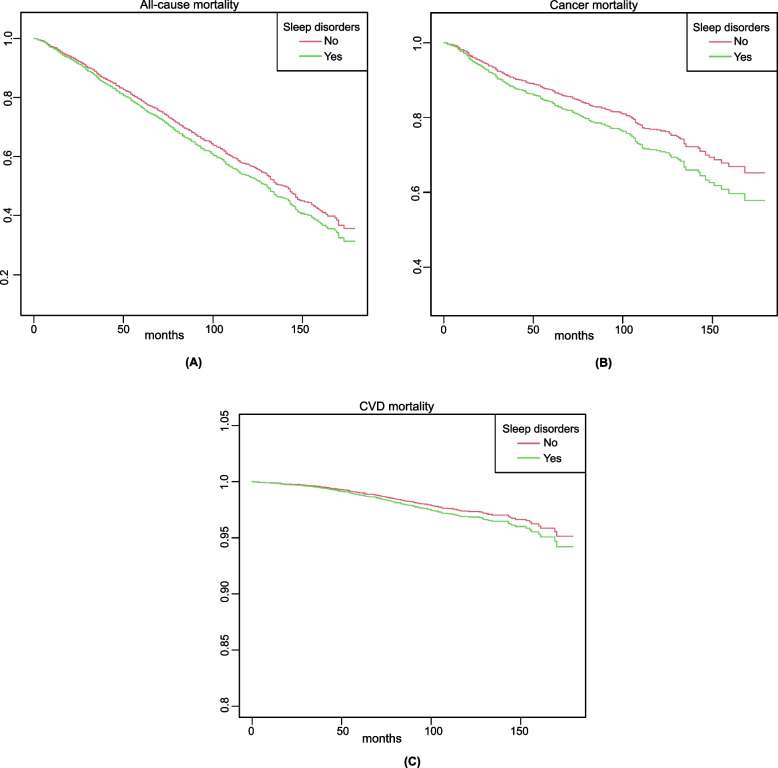
Multiple adjusted Kaplan-Meier survival curves for mortality outcomes in the total population. Kaplan-Meier survival curves for **A** all-cause mortality, **B** cancer mortality, and **C** CVD mortality in the total population by sleep disorder status. The analysis were adjusted for gender, age, race, education levels, BMI, smoking history, alcoholic drinking, and coronary heart disease. BMI, body mass index; CVD, cardiovascular disease

In the whole group, after adjusting for other potential determinants, Cox regression analysis demonstrated that sleep disorders significantly increased the risks of all-cause mortality, cancer mortality, and CVD mortality (Table 2). In Model I, the multivariable-adjusted hazard ratio (HR) (95% confidence intervals (CIs) was 1.27 (1.11, 1.45) for all-cause mortality, 1.33 (1.06, 1.67) for cancer mortality, and 1.47 (1.13, 1.92) for CVD mortality. In Model II, the multivariable-adjusted HRs (95% CIs) were 1.23 (1.06, 1.42) for all-cause mortality, 1.30 (1.02, 1.66) for cancer mortality, and 1.35 (1.02, 1.80) for CVD mortality, The *P* values for all multivariable-adjusted models are below 0.05. Fig. 2 demonstrates the multiple adjusted Kaplan-Meier curves for mortality outcomes by sleep disorder status. In individuals with and without sleep disorders, the adjusted median survival time was 130 months and 140 months, the 10-year survival rate was 53.3 and 57.1% for all-cause mortality. For cancer deaths, the adjusted median survival time was 131 months and 151 months, the 10-year survival rate was 71.1 and 76.7%. For CVD deaths, the 10-year survival rate was 96.9 and 97.4% respectively.

After the total group was stratified by gender, we observed that sleep disorders significantly increased the risks of all-cause mortality, cancer mortality, and CVD mortality among males, but not among female individuals (Table 2). In male participants, the multivariable-adjusted HRs (95% CIs) in Model I were 1.52 (1.27, 1.81) for all-cause mortality, 1.88 (1.42, 2.49) for cancer mortality and 1.49 (1.05, 2.12) for CVD mortality. In Model II, the multivariable-adjusted HRs (95% CIs) were 1.47 (1.22, 1.77) for all-cause mortality, 1.82 (1.34, 2.46) for cancer mortality, and 1.49 (1.03, 2.16) for CVD mortality. The *P* values for all multivariable-adjusted models are below 0.05.

### Stratified analysis for sleep disorders and mortalities

**Fig. 3:**
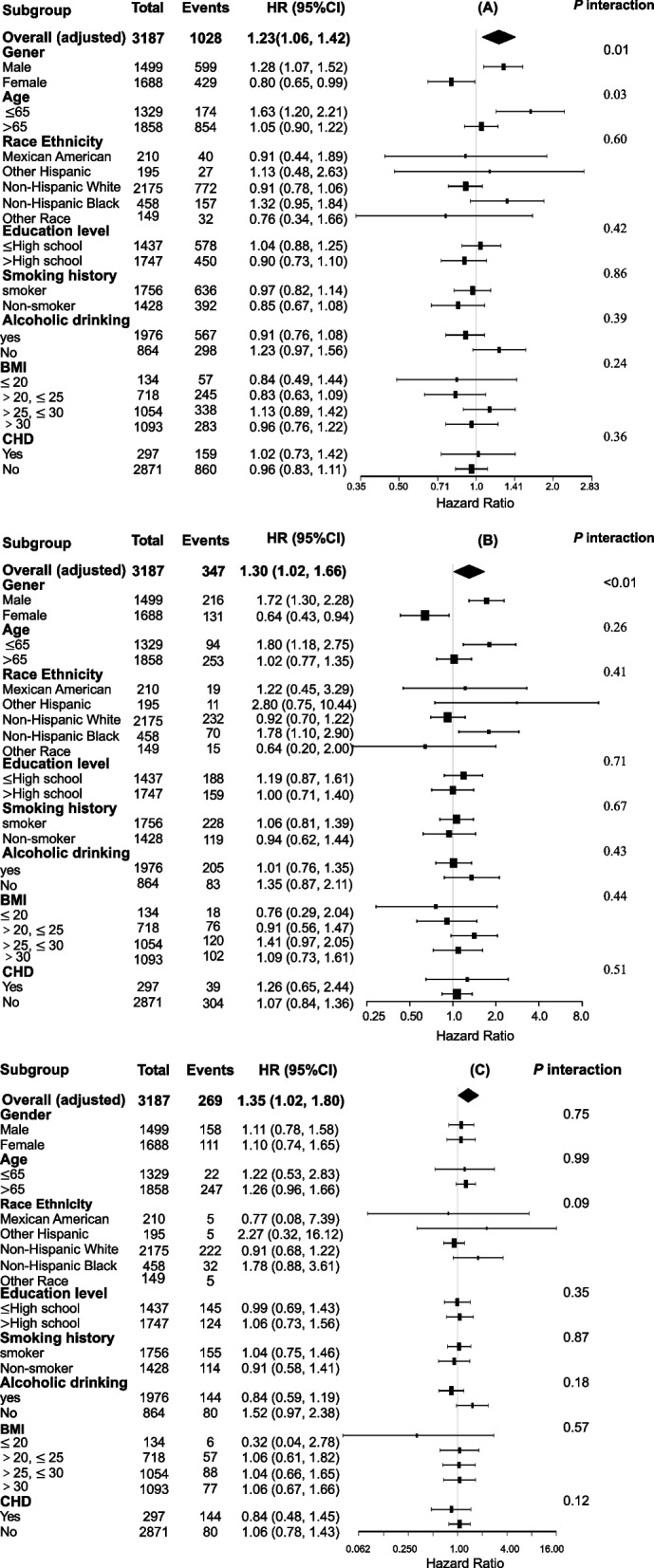
Forest plots for the stratified analysis in the total population. The forest plots shows the analysis of sleep disorders-mortality associations stratified by characteristics in the total population for **A** all-cause mortality, **B** cancer mortality, and **C** CVD mortality. The analysis were adjusted for gender, age, race, BMI, education levels, smoking history, alcoholic drinking, and coronary heart disease. BMI, body mass index; CVD, cardiovascular disease; HR, hazard ratio; CI, confidence interval

In the total group, stratified analyses were conducted to determine if sleep disorder status and mortalities differed by gender, age, race ethnicity, education level, smoking history, alcoholic drinking, BMI, and coronary heart disease. The results were reliable, without statistically significant interaction for mortalities, except for gender and age for all-cause mortality (Fig. 3A, B, and C).

Among male participants, stratified analysis suggested no potential modifiers in the relationship between sleep disorder status and all-cause mortality, cancer mortality, and CVD mortality (Supplementary Fig. 1A, B, and C). Of note, no significant interactions were found between sleep disorder status and the stratum variable (*P* interaction > 0.05).

### Sleep disorders with long-term mortalities

We further excluded cancer individuals who died within 2 years of follow-up to investigate whether sleep disorders are associated with long-term outcomes among the total population and male subgroup. In the whole group, sleep disorders still correlated to higher long-term all-cause mortality, with a multivariable-adjusted HRs (95% CIs) 1.24 (1.07, 1.45) in model I, 1.20 (1.02, 1.42) in model II. But For the long-term cancer mortality, the sleep disorder-mortality association was only significant in model I with a 1.32-fold (1.00, 1.74) risk. For the long-term CVD mortality, participants with sleep disorders were associated with a 1.5-fold (1.12–2.02) risk in model I, and 1.45-fold (1.06–1.99) risk in model II than those without (Supplementary Table 1).

In male individuals, having a sleep disorder remained a significant predictor of increased long-term all-cause mortality with HRs (95% CIs) 1.43 (1.17, 1.75) in model I and 1.41 (1.13, 1.75) in model II. For long-term cancer mortality, sleep disorders were associated with 1.88-fold (1.34, 2.65) risk in model I and 1.92-fold (1.32, 2.79) risk in model II compared to individuals without sleep disorders (Supplementary Table 1).

### Sensitive analysis

For further validation of the outcomes, we used the data (NHANES 2005–2006, 2007–2008, 2009–2010, 2011–2012, 2013–2014) to explore the association between mortalities and sleep disorders (defined by another specific question SLQ060: “Have you ever been told by a doctor or other health professional that you have a sleep disorder?” and those participants responding “Yes” were considered to have sleep disorders) by the multivariable Cox proportional hazards models after excluding the individuals who died within 2 years of follow-up. The results were consistent with the results of the main analysis. Supplementary Table 2 and Supplementary Table 3 Show the data Baseline characteristics and the results of multivariable Cox proportional hazards models respectively. In addition, considering the possibility that the estimates for the cause-specific mortality risks could be biased by participants dying from non-cancer or non-CVD events that are incorrectly considered censoring events, we also estimated cancer and CVD mortality by competing risks models. After multiple adjustments for covariates including age, gender (only for overall), race/ethnicity, education level, BMI, smoking history, alcohol drinking history, and coronary heart disease, competing risk models showed that sleep disorders significantly increased the risks of cancer mortality (HR, 1.35; 95% CI, 1.06–1.73), CVD mortality (HR, 1.36; 95% CI, 1.02–1.82), and long-term CVD mortality (HR, 1.44; 95% CI, 1.05–1.98) in total population. In male participants, sleep disorders also significantly increased the risks of cancer mortality (HR, 1.95; 95% CI, 1.44–2.66), and long-term cancer mortality (HR, 1.96; 95% CI, 1.35–2.86) (Supplementary Table 4).

## Discussion

In this study of 3187 participants with cancer from the NHANES 2005–2016 cohorts, after a median follow-up time of 83.0 (interquartile range: 51.0–122.0) months and adjusting for covariates, multivariable Cox proportional hazards models showed that sleep disorders were associated with a higher risk of all-cause mortality, cancer mortality, and CVD mortality. Intriguingly, after the total group was stratified by gender, The high HRs were observed in men, not in women. The results were consistent after individuals who died within 2 years of follow-up were excluded, indicating that sleep disorders also correlate to higher long-term mortality in cancer patients. Sensitive analysis did not observe any profound changes, which suggested that our results were robust.

Our result demonstrated that sleep disorders were associated with a higher risk of all-cause and CVD mortality. Despite some inconsistencies [5, 23], increasing evidence has demonstrated the association between sleep disorders and elevated risks of all-cause and disease-specific mortality in general populations or non-cancer patients [8, 10, 24–26]. For example, a prospective cohort analysis included a general population more than 18 years old from the NHANES 2005–2018 reported the association between self-reported sleep disturbance and higher risk of all-cause mortality with an HR of 1.17 (95% CI, 1.04–1.32) in a fully-adjusted model [24]. In another prospective cohort study, sleep disorder was also significantly associated with total mortality with an HR and 95% CI 1.49 (1.28–1.72) without joint effects with depression [8]. Similar associations were also supported by a few meta-analyses [27, 28]. Such a positive correlation between sleep disorders and high risks for all-cause and CVD mortality could partly be explained by a previous study reporting that sleep disorders were associated with the elevation of systemic inflammation markers including C-reactive protein and interleukin-6 [29], which contribute to the risk of a wide spectrum of medical conditions and predict cardiovascular events [30, 31]. The data about such a relationship in cancer patients are limited. A study focusing on a general adult population in US adults from NHANES 2005–2014 reported that sleep complaints were associated with all-cause and heart disease mortality among the subgroup with cancer [32]. This was consistent with our result and indicated the higher mortality in all-cause and CVD should be aware in cancer patients with sleep disorders.

Our study demonstrated that sleep disorders were correlated to a higher risk of cancer mortality in cancer patients. Evidence for the correlation between sleep disorders and cancer mortality is rare and controversial. For the general population, a study of 380,055 general participants from the UK Biobank demonstrated that poor sleep was associated with increased risk for total cancer mortality [9]. However, another large prospective cohort study free of pre-existing disease drew an opposite conclusion that sleep was not correlated to death due to cancer [23]. Although the relative risk was substantial in our study, the absolute risk difference observed was small, therefore, it might be underpowered for some studies to detect such effects. Sleep disorders in cancer patients are common and may have distinct backgrounds compared with those in general populations, due to the differences in risk factors, susceptibility, or cancer-specific life events. There is a close connection between the circadian clock and endoplasmic reticulum stress signaling, oncogenesis, and cancer progress. The sleep-wake rhythm is subjected to circadian control and recognized to be a marker rhythm of the circadian system [33]. However, the clinical evidence for the association between sleep disorders and cancer mortality in cancer patients is rare and needs to be further investigated. In patients with colorectal cancer, sleep deficiency was reported to be associated with a 54% increase in colorectal cancer mortality [34]. Another prospective study of 1175 patients with stage III colon cancer in the CALGB/SWOG 80702 randomized trial also demonstrated that sleep problems were associated with increased mortality [35]. Collins et al. reported that sleep duration is linked to mortality in advanced cancer patients [16]. A retrospective study in breast cancer women indicated that sleep disorders were significantly associated with early mortality [15], but the study deals with all-cause mortality and lacks information on the actual cause of death. The finding above underlines the need to integrate sleep health more effectively as a necessary component of cancer care.

Sleep disorders could appear before, during, and after cancer diseases. Considering that sleep problems could be merely a concomitant symptom of a health situation rather than a risk factor, our study further excluded participants who died within 2 years of follow-up and demonstrated that sleep disorders also correlate to higher long-term mortality in total cancer patients and the male subgroup. Though the causal relationships between sleep disorders and mortality in the cancer population could not be inferred due to the cross-sectional design, the correlation between sleep disorders and increased long-term mortality could be relatively more convincing to imply a causal relationship between sleep disturbance and elevated mortality risk. Our result was consistent with a study demonstrating that subjective sleep problems are associated with poor clinical outcomes in treatment-naive metastatic colorectal cancer patients [36]. However, our result needs to be further elucidated in further studies with repeated recordings of sleep profiles as well as considering more covariates including detailed cancer information.

The association between sleep disorders and the elevated mortality risks in our result was significant in men, not in women. Similar to our finding, Rod et al. reported that men with sleep disturbances, not women, were correlated to an increased overall mortality risk in a general population. Men reporting lying awake most of the night (HR 1.39, 95% CI: 1.00, 1.93) or sleeping badly at night (HR 1.56, 95% CI: 0.96, 2.51) were at a higher risk of cancer mortality [37]. Another study showed a notably higher risk of all-cause mortality in men, but not in women, with short sleep duration and insomnia independent of comorbid conditions frequently associated with mortality [38]. Our result was also supported by some recent prospective cohort studies in a general population from NHANES indicating the difference in the sleep disorders-mortality association in men and women [8, 24]. Such gender differences may partly be explained by the role of oxidative stress, which has been involved in sleep deprivation-related impairments [39]. Women appear to be less susceptible to oxidative stress [40], the difference in antioxidant levels between men and women may be an important factor. Still both the underlying biological and psychosocial mechanisms need to be elucidated.

The strength of our study includes the use of a large nationally non-institutionalized US population with cancer, reliable mortality records, long period of follow-up, multivariable Cox regression models in the statistical analysis, and the competing risk models introduced in sensitive analysis considering the deaths with other causes as competing risks, which all enhanced the reliability and precision of our findings. Additionally, although self-report sleep disorder was considered an imprecise measure, as a comprehensive notion of sleep status, it remains a crucial indicator for sleep health, as it may mirror the presence of sleep detriment hard to identify and assess. We analyzed the correlation between sleep disorders and long-term mortalities, which could be more convincing to infer a causal relationship between sleep disorders and mortalities.

However, our analysis had certain limitations. First, self-reported sleep disorders without objective sleep measures might induce bias. Second, sleep behaviors assessed in baseline only could not reflect the long-term status precisely. Third, limited by the questionnaires of NHANES, we could not explore the relationships between sleep disorder symptoms, such as insomnia, restless legs syndrome, and mortality. Additionally, since the number of deaths was limited, specific cancer analysis was lacking. More large-scale studies, including clinical trials, are needed. Last, due to the cross-sectional design, the causal relationships between sleep disorders and mortality in the population of cancer should not be inferred.

## Conclusion

In conclusion, our study indicated that sleep disorders were associated with a higher risk of all-cause mortality, cancer mortality, and CVD mortality, as well as long-term mortality in cancer patients. Our finding underlies the importance of screening for sleep disorders for all cancer survivors—which is recommended by the NCCN, to reduce complications, and improve patient’s quality of life and survival. Male individuals may be particularly vulnerable and could benefit from more frequent screening.

### Supplementary Information

** Additional file 1:** Supplementary Material 1.

** Additional file 2: **Supplementary Material 2.

## Data Availability

No datasets were generated or analysed during the current study.

## Abbreviations

CVD: Cardiovascular disease
NHANES: National Health and Nutrition Examination Survey
NDI: National Death Index
CAPI: Computer-assisted personal interview
MEC: Mobile examination center
ICD-10: The 10th international classification of diseases
BMI: Body mass index
SD: Standard deviation
HR: Hazard ratio
CIs: Confidence intervals

## References

[1] Sateia MJ (2014). International classification of sleep disorders-third edition: highlights and modifications. Chest.

[2] Consensus Conference P, Watson NF, Badr MS, Belenky G, Bliwise DL, Buxton OM, Buysse D, Dinges DF, Gangwisch J, Grandner MA (2015). Joint Consensus statement of the American Academy of sleep medicine and Sleep Research Society on the recommended amount of sleep for a healthy adult: methodology and discussion. Sleep.

[3] Summa KC, Turek FW (2014). Chronobiology and obesity: interactions between circadian rhythms and energy regulation. Adv Nutr.

[4] Holmer BJ, Lapierre SS, Jake-Schoffman DE, Christou DD (2021). Effects of sleep deprivation on endothelial function in adult humans: a systematic review. Geroscience.

[5] Bertisch SM, Pollock BD, Mittleman MA, Buysse DJ, Bazzano LA, Gottlieb DJ, et al. Insomnia with objective short sleep duration and risk of incident cardiovascular disease and all-cause mortality: sleep heart health study. Sleep. 2018;41(6) 10.1093/sleep/zsy047. 10.1093/sleep/zsy047 PMC5995202 29522193

[6] Yin J, Jin X, Shan Z, Li S, Huang H, Li P, et al. Relationship of sleep duration with all-cause mortality and cardiovascular events: a systematic review and dose-response Meta-analysis of prospective cohort studies. J Am Heart Assoc. 2017;6(9) 10.1161/JAHA.117.005947. 10.1161/JAHA.117.005947 PMC5634263 28889101

[7] Javaheri S, Redline S (2017). Insomnia and risk of cardiovascular disease. Chest.

[8] Li W, Chen D, Ruan W, Peng Y, Lu Z, Wang D (2022). Associations of depression, sleep disorder with total and cause-specific mortality: a prospective cohort study. J Affect Disord.

[9] Huang BH, Duncan MJ, Cistulli PA, Nassar N, Hamer M, Stamatakis E (2022). Sleep and physical activity in relation to all-cause, cardiovascular disease and cancer mortality risk. Br J Sports Med.

[10] Huyett P, Siegel N, Bhattacharyya N (2021). Prevalence of sleep disorders and association with mortality: results from the NHANES 2009-2010. Laryngoscope.

[11] Siegel RL, Miller KD, Fuchs HE, Jemal A (2021). Cancer statistics, 2021. CA Cancer J Clin.

[12] Slade AN, Waters MR, Serrano NA (2020). Long-term sleep disturbance and prescription sleep aid use among cancer survivors in the United States. Support Care Cancer.

[13] Pluquet O, Dejeans N, Chevet E (2014). Watching the clock: endoplasmic reticulum-mediated control of circadian rhythms in cancer. Ann Med.

[14] Mogavero MP, DelRosso LM, Fanfulla F, Bruni O, Ferri R (2021). Sleep disorders and cancer: state of the art and future perspectives. Sleep Med Rev.

[15] Bach L, Kalder M, Kostev K (2021). Depression and sleep disorders are associated with early mortality in women with breast cancer in the United Kingdom. J Psychiatr Res.

[16] Collins KP, Geller DA, Antoni M, Donnell DM, Tsung A, Marsh JW, Burke L, Penedo F, Terhorst L, Kamarck TW (2017). Sleep duration is associated with survival in advanced cancer patients. Sleep Med.

[17] Curtin LR, Mohadjer LK, Dohrmann SM, Kruszon-Moran D, Mirel LB, Carroll MD, et al. National Health and nutrition examination survey: sample design, 2007-2010. Vital Health Stat 2. 2013(160):1–23. 25090039

[18] Li C, Ford ES, Zhao G, Tsai J, Balluz LS (2012). A comparison of depression prevalence estimates measured by the patient health questionnaire with two administration modes: computer-assisted telephone interviewing versus computer-assisted personal interviewing. Int J Public Health.

[19] Deng MG, Liu F, Liang Y, Chen Y, Nie JQ, Chai C, Wang K (2023). Associations of serum zinc, copper, and selenium with sleep disorders in the American adults: data from NHANES 2011-2016. J Affect Disord.

[20] Rahman HH, Niemann D, Yusuf KK (2022). Association of urinary arsenic and sleep disorder in the US population: NHANES 2015-2016. Environ Sci Pollut Res Int.

[21] Liu J, Rehm CD, Onopa J, Mozaffarian D (2020). Trends in diet quality among youth in the United States, 1999-2016. JAMA.

[22] Liesbeth C, de Wreede MF. Hein putter: mstate: an R package for the analysis of competing risks and multi-state models. J Stat Softw. 2011;38(7) 10.18637/jss.v038.i07.

[23] Rod NH, Kumari M, Lange T, Kivimaki M, Shipley M, Ferrie J (2014). The joint effect of sleep duration and disturbed sleep on cause-specific mortality: results from the Whitehall II cohort study. PLoS One.

[24] Hou X, Hu J, Wang E, Wang J, Song Z, Hu J, Shi J, Zhang C (2023). Self-reported sleep disturbance is an independent predictor of all-cause mortality and respiratory disease mortality in US adults: a population-based prospective cohort study. Int J Public Health.

[25] Luskin MR, Cronin AM, Owens RL, DeAngelo DJ, Stone RM, Wadleigh M, Steensma DP, Abel GA (2017). Self-reported sleep disturbance and survival in myelodysplastic syndromes. Br J Haematol.

[26] Zhou T, Yuan Y, Xue Q, Li X, Wang M, Ma H, Heianza Y, Qi L (2022). Adherence to a healthy sleep pattern is associated with lower risks of all-cause, cardiovascular and cancer-specific mortality. J Intern Med.

[27] Ge L, Guyatt G, Tian J, Pan B, Chang Y, Chen Y, Li H, Zhang J, Li Y, Ling J (2019). Insomnia and risk of mortality from all-cause, cardiovascular disease, and cancer: systematic review and meta-analysis of prospective cohort studies. Sleep Med Rev.

[28] Liu TZ, Xu C, Rota M, Cai H, Zhang C, Shi MJ, Yuan RX, Weng H, Meng XY, Kwong JS (2017). Sleep duration and risk of all-cause mortality: a flexible, non-linear, meta-regression of 40 prospective cohort studies. Sleep Med Rev.

[29] Irwin MR, Olmstead R, Carroll JE (2016). Sleep disturbance, sleep duration, and inflammation: a systematic review and Meta-analysis of cohort studies and experimental sleep deprivation. Biol Psychiatry.

[30] Ridker PM, Buring JE, Cook NR, Rifai N (2003). C-reactive protein, the metabolic syndrome, and risk of incident cardiovascular events: an 8-year follow-up of 14 719 initially healthy American women. Circulation.

[31] Ridker PM, Rifai N, Rose L, Buring JE, Cook NR (2002). Comparison of C-reactive protein and low-density lipoprotein cholesterol levels in the prediction of first cardiovascular events. N Engl J Med.

[32] Wang Q, Hu S, Pan NC, Zhang T, Ren L, Wang Y (2023). Association of sleep complaints with all-cause and heart disease mortality among US adults. Front Public Health.

[33] Hofstra WA, de Weerd AW (2008). How to assess circadian rhythm in humans: a review of literature. Epilepsy Behav.

[34] Xiao Q, Arem H, Pfeiffer R, Matthews C. Prediagnosis sleep duration, napping, and mortality among colorectal Cancer survivors in a large US cohort. Sleep. 2017;40(4) 10.1093/sleep/zsx010. 10.1093/sleep/zsx010 PMC5806565 28329353

[35] Lee S, Ma C, Shi Q, Meyers J, Kumar P, Couture F, Kuebler P, Krishnamurthi S, Lewis D, Tan B (2023). Sleep and cancer recurrence and survival in patients with resected stage III colon cancer: findings from CALGB/SWOG 80702 (Alliance). Br J Cancer.

[36] Innominato PF, Spiegel D, Ulusakarya A, Giacchetti S, Bjarnason GA, Levi F, Palesh O (2015). Subjective sleep and overall survival in chemotherapy-naive patients with metastatic colorectal cancer. Sleep Med.

[37] Rod NH, Vahtera J, Westerlund H, Kivimaki M, Zins M, Goldberg M, Lange T (2011). Sleep disturbances and cause-specific mortality: results from the GAZEL cohort study. Am J Epidemiol.

[38] Vgontzas AN, Liao D, Pejovic S, Calhoun S, Karataraki M, Basta M, Fernandez-Mendoza J, Bixler EO (2010). Insomnia with short sleep duration and mortality: the Penn State cohort. Sleep.

[39] Atrooz F, Salim S (2020). Sleep deprivation, oxidative stress and inflammation. Adv Protein Chem Struct Biol.

[40] Borras C, Sastre J, Garcia-Sala D, Lloret A, Pallardo FV, Vina J (2003). Mitochondria from females exhibit higher antioxidant gene expression and lower oxidative damage than males. Free Radic Biol Med.

