# Supplementary material for: Associations of sleep disorders with all-cause and cause-specific mortality in cancer survivors: a cross-sectional analysis of the NHANES 2005–2016
Source: BMC Psychiatry. 2024 Feb 12;24:118. doi: 10.1186/s12888-024-05589-3 (PMC10863252; doi:10.1186/s12888-024-05589-3)
Supplement: Supplementary file 1 — Additional file 1: Supplementary Material 1. [file 12888_2024_5589_MOESM1_ESM.docx]

| Supplementary Table 1 The relationship between sleep disorders and long-term mortality among cancer participants, NHANES(2005-2016) | | | | | | | | |
| --- | --- | --- | --- | --- | --- | --- | --- | --- |
| Outcomes | **Non-adjusted Model** | |  | **Model I** | |  | **Model II** | |
|  | HR (95% CI) | *P*-value |  | HR (95% CI) | *P*-value |  | HR (95% CI) | *P*-value |
| Sleep disorders | | | | | | | | |
| Long-term all-cause mortality No. of deaths/participants (824/2983) | | | | | | | | |
| Total | | | | | | | | |
| No | 1(reference) |  |  | 1(reference) |  |  | 1(reference) |  |
| Yes | 1.00 (0.85, 1.16) | 0.95 |  | 1.24 (1.07, 1.45) | <0.01 |  | 1.20 (1.02, 1.42) | <0.05 |
| Male | | | | | | | | |
| No | 1(reference) |  |  | 1(reference) |  |  | 1(reference) |  |
| Yes | 1.16 (0.95, 1.42) | 0.14 |  | 1.43 (1.17, 1.75) | <0.001 |  | 1.41 (1.13, 1.75) | <0.01 |
| Female |  |  |  |  |  |  |  |  |
| No | 1(reference) |  |  | 1(reference) |  |  | 1(reference) |  |
| Yes | 0.82 (0.65, 1.03) | 0.09 |  | 1.04 (0.83, 1.32) | 0.72 |  | 1.01 (0.79, 1.30) | 0.93 |
| Long-term cancer mortality No. of deaths/ participants (252/2983) | | | | | | | | |
| Total | | | | | | | | |
| No | 1(reference) |  |  | 1(reference) |  |  | 1(reference) |  |
| Yes | 1.16 (0.88, 1.52) | 0.29 |  | 1.32 (1.00, 1.74) | <0.05 |  | 1.31 (0.98, 1.77) | 0.07 |
| Male |  |  |  |  |  |  |  |  |
| No | 1(reference) |  |  | 1(reference) |  |  | 1(reference) |  |
| Yes | 1.67 (1.19, 2.35) | <0.01 |  | 1.88 (1.34, 2.65) | <0.001 |  | 1.92 (1.32, 2.79) | <0.001 |
| Female |  |  |  |  |  |  |  |  |
| No | 1(reference) |  |  | 1(reference) |  |  | 1(reference) |  |
| Yes | 0.66 (0.42, 1.04) | 0.08 |  | 0.77 (0.49, 1.21) | 0.26 |  | 0.75 (0.46, 1.22) | 0.24 |
| Long-term CVD mortality No. of deaths/ participants (225/2983) | | | | | | | | |
| Total | | | | | | | | |
| No | 1(reference) |  |  | 1(reference) |  |  | 1(reference) |  |
| Yes | 1.09 (0.81, 1.47) | 0.55 |  | 1.50 (1.12, 2.02) | <0.01 |  | 1.45 (1.06, 1.99) | <0.05 |
| Male |  |  |  |  |  |  |  |  |
| No | 1(reference) |  |  | 1(reference) |  |  | 1(reference) |  |
| Yes | 1.01 (0.68, 1.49) | 0.96 |  | 1.40 (0.94, 2.08) | 0.09 |  | 1.39 (0.92, 2.11) | 0.12 |
| Female |  |  |  |  |  |  |  |  |
| No | 1(reference) |  |  | 1(reference) |  |  | 1(reference) |  |
| Yes | 1.22 (0.78, 1.91) | 0.40 |  | 1.64 (1.04, 2.59) | <0.05 |  | 1.60 (0.97, 2.65) | 0.07 |

Model I: adjusted for age, gender (only for overall)

Model II: adjusted for age, gender (only for overall), education levels, race, smoking history, alcoholic drinking, BMI, coronary heart disease

NHANES, National Health and Nutrition Examination Survey; BMI, body mass index; CVD, cardiovascular disease; HR, hazard ratio; CI, confidence interval

| Supplementary Table 2 Baseline characteristics of participants stratified by sleep disorder status NHANES 2005-2014 | | | | |
| --- | --- | --- | --- | --- |
| Characteristics | Total  No (n=2480) | Sleep disorders | | *P*-value |
|  |  | No (n=2186) | Yes (n=294) |  |
| Age, mean ± sd, y | 64.97 ± 14.65 | 65.42 ± 14.77 | 61.65 ± 13.21 | <0.001 |
| Gender |  |  |  | 0.018 |
| Male | 1143 (46.09%) | 989 (45.24%) | 154 (52.56%) |  |
| Female | 1337 (53.91%) | 1197 (54.76%) | 139 (47.44%) |  |
| Race/ethnicity |  |  |  | 0.957 |
| Mexican american | 161 (6.49%) | 144 (6.59%) | 17 (5.80%) |  |
| Other hispanic | 132 (5.32%) | 116 (5.31%) | 16 (5.46%) |  |
| Non-hispanic white | 1735 (69.96%) | 1527 (69.85%) | 207 (70.65%) |  |
| Non-hispanic black | 352 (14.19%) | 309 (14.14%) | 43 (14.68%) |  |
| Other race - including multi-racial | 100 (4.03%) | 90 (4.12%) | 10 (3.41%) |  |
| Education |  |  |  | 0.047 |
| Less than 9th grade | 255 (10.29%) | 223 (10.20%) | 32 (10.92%) |  |
| 9-11th grade | 321 (12.95%) | 291 (13.31%) | 30 (10.24%) |  |
| High school graduate | 553 (22.32%) | 500 (22.87%) | 53 (18.09%) |  |
| Some college or aa degree | 694 (28.01%) | 593 (27.13%) | 101 (34.47%) |  |
| College graduate or above | 655 (26.43%) | 577 (26.40%) | 77 (26.28%) |  |
| Smoking history |  |  |  | <0.001 |
| Smoker | 1349 (54.44%) | 1160 (53.11%) | 188 (64.16%) |  |
| Non-smoker | 1129 (45.56%) | 1024 (46.89%) | 105 (35.84%) |  |
| Alcoholic drinking |  |  |  | 0.060 |
| Yes | 1540 (69.81%) | 1338 (69.11%) | 201 (74.72%) |  |
| No | 666 (30.19%) | 598 (30.89%) | 68 (25.28%) |  |
| BMI(kg/m2) | 28.95 ± 6.44 | 28.44 ± 6.09 | 32.70 ± 7.59 | <0.001 |
| Coronary heart disease |  |  |  | 0.017 |
| Yes | 207 (8.40%) | 172 (7.87%) | 35 (11.95%) |  |
| No | 2257 (91.60%) | 2001 (91.54%) | 255 (87.03%) |  |

NHANES, National Health and Nutrition Examination Survey; BMI, body mass index

| Supplementary Table 3 The relationship between sleep disorders and long-term mortality among cancer participants, NHANES(2005-2014) | | | | | | | | |
| --- | --- | --- | --- | --- | --- | --- | --- | --- |
| Outcomes | **Non-adjusted Model** | |  | **Model I** | |  | **Model II** | |
|  | HR (95% CI) | *P*-value |  | HR (95% CI) | *P*-value |  | HR (95% CI) | *P*-value |
| Sleep disorders (SLQ 060) | | | | | | | | |
| Long-term all-cause mortality No. of deaths/participants (783/2480) | | | | | | | | |
| No | 1(reference) |  |  | 1(reference) |  |  | 1(reference) |  |
| Yes | 0.90 (0.71, 1.13) | 0.36 |  | 1.28 (1.01, 1.63) | 0.04 |  | 1.41 (1.10, 1.82) | 0.007 |
| Long-term cancer mortality No. of deaths/ participants (238/2480) | | | | | | | | |
| No | 1(reference) |  |  | 1(reference) |  |  | 1(reference) |  |
| Yes | 1.10 (0.74, 1.64) | 0.6388 |  | 1.31 (0.87, 1.95) | 0.1932 |  | 1.57 (1.02, 2.41) | 0.038 |
| Long-term CVD mortality No. of deaths/ participants (215/2480) | | | | | | | | |
| No | 1(reference) |  |  | 1(reference) |  |  | 1(reference) |  |
| Yes | 0.76 (0.47, 1.24) | 0.2687 |  | 1.31 (0.80, 2.15) | 0.2834 |  | 1.31 (0.77, 2.20) | 0.31 |

Model I: adjusted for age, gender

Model II: adjusted for age, gender, education levels, race, smoking history, alcoholic drinking, BMI, coronary heart disease

NHANES, National Health and Nutrition Examination Survey; BMI, body mass index; CVD, cardiovascular disease; HR, hazard ratio; CI, confidence interval

| Supplementary Table 4 Competing risks models for specific-cause mortality and long-term specific-cause mortality by sleep disorder status | | | | |
| --- | --- | --- | --- | --- |
| **Outcomes** | **Cancer mortality** | **CVD mortality** | **Long-term Cancer mortality** | **Long-term CVD mortality** |
| **Sleep disorder status** | HR (95% CI) | HR (95% CI) | HR (95% CI) | HR (95% CI) |
| **Total** (No. of deaths/ participants ) | (347/3187) | (269/3187) | (252/2983) | (225/2983) |
| No | 1 (reference) | 1 (reference) | 1 (reference) | 1 (reference) |
| Yes | 1.35 (1.06, 1.73) | 1.36 (1.02, 1.82) | 1.34 (0.99, 1.80) | 1.44 (1.05, 1.98) |
| **Male** |  |  |  |  |
| No | 1 (reference) | 1 (reference) | 1 (reference) | 1 (reference) |
| Yes | 1.95 (1.44, 2.66) | 1.47 (1.00, 2.16) | 1.96 (1.35, 2.86) | 1.40 (0.92, 2.14) |
| **Female** |  |  |  |  |
| No | 1 (reference) | 1 (reference) | 1 (reference) | 1 (reference) |
| Yes | 0.74 (0.48, 1.13) | 1.25 (0.79, 1.97) | 0.75 (0.45, 1.23) | 1.59 (0.95, 2.63) |
| CVD, cardiovascular disease; HR, hazard ratio; CI, confidence interval | | | | |
