# Supplementary material for: Associations of sleep disorders with all-cause and cause-specific mortality in cancer survivors: a cross-sectional analysis of the NHANES 2005–2016
Source: BMC Psychiatry. 2024 Feb 12;24:118. doi: 10.1186/s12888-024-05589-3 (PMC10863252; doi:10.1186/s12888-024-05589-3)
Supplement: Supplementary file 2 — Additional file 2: Supplementary Material 2. [file 12888_2024_5589_MOESM2_ESM.pptx]

## Slide 1
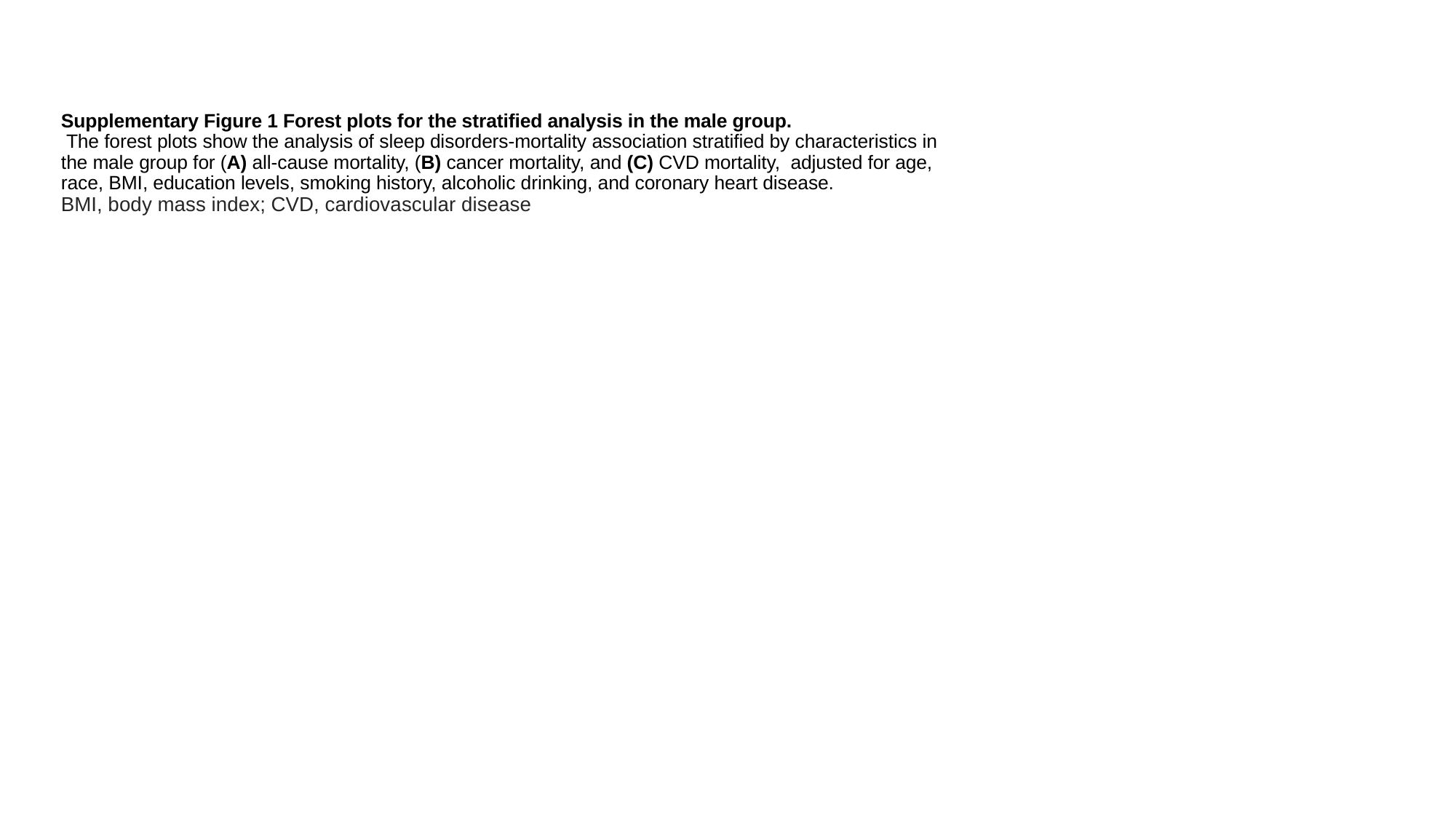

# Supplementary Figure 1 Forest plots for the stratified analysis in the male group. The forest plots show the analysis of sleep disorders-mortality association stratified by characteristics in the male group for (A) all-cause mortality, (B) cancer mortality, and (C) CVD mortality, adjusted for age, race, BMI, education levels, smoking history, alcoholic drinking, and coronary heart disease. BMI, body mass index; CVD, cardiovascular disease

## Slide 2
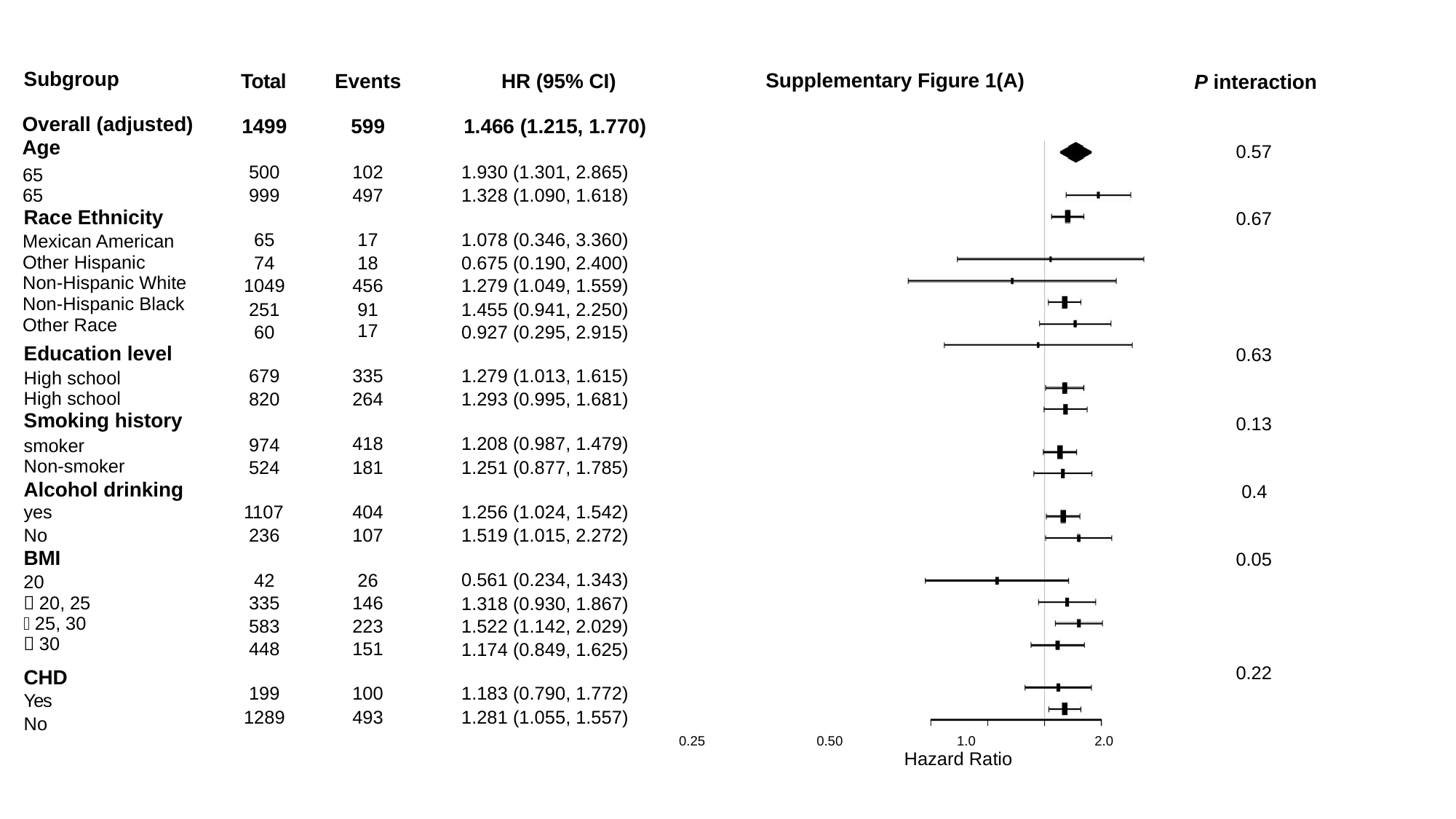

Supplementary Figure 1(A)
Subgroup
Total
1499
Events
599
HR (95% CI)
1.466 (1.215, 1.770)
P interaction
Overall (adjusted)
Age
0.57
500
999
102
497
1.930 (1.301, 2.865)
1.328 (1.090, 1.618)
Race Ethnicity
0.67
65
74
1049
251
60
1.078 (0.346, 3.360)
0.675 (0.190, 2.400)
1.279 (1.049, 1.559)
1.455 (0.941, 2.250)
0.927 (0.295, 2.915)
17
18
456
91
Mexican American
Other Hispanic
Non-Hispanic White
Non-Hispanic Black
Other Race
17
Education level
Smoking history
0.63
0.13
0.4
679
820
974
524
335
264
1.279 (1.013, 1.615)
1.293 (0.995, 1.681)
418
181
1.208 (0.987, 1.479)
1.251 (0.877, 1.785)
smoker
Non-smoker
Alcohol drinking
yes
No
BMI
1107
236
404
107
1.256 (1.024, 1.542)
1.519 (1.015, 2.272)
0.05
42
26
0.561 (0.234, 1.343)
1.318 (0.930, 1.867)
1.522 (1.142, 2.029)
1.174 (0.849, 1.625)
335
583
448
146
223
151
＞30
0.22
CHD
Yes
No
199
1289
100
493
1.183 (0.790, 1.772)
1.281 (1.055, 1.557)
0.25
0.50
1.0
2.0
Hazard Ratio

## Slide 3
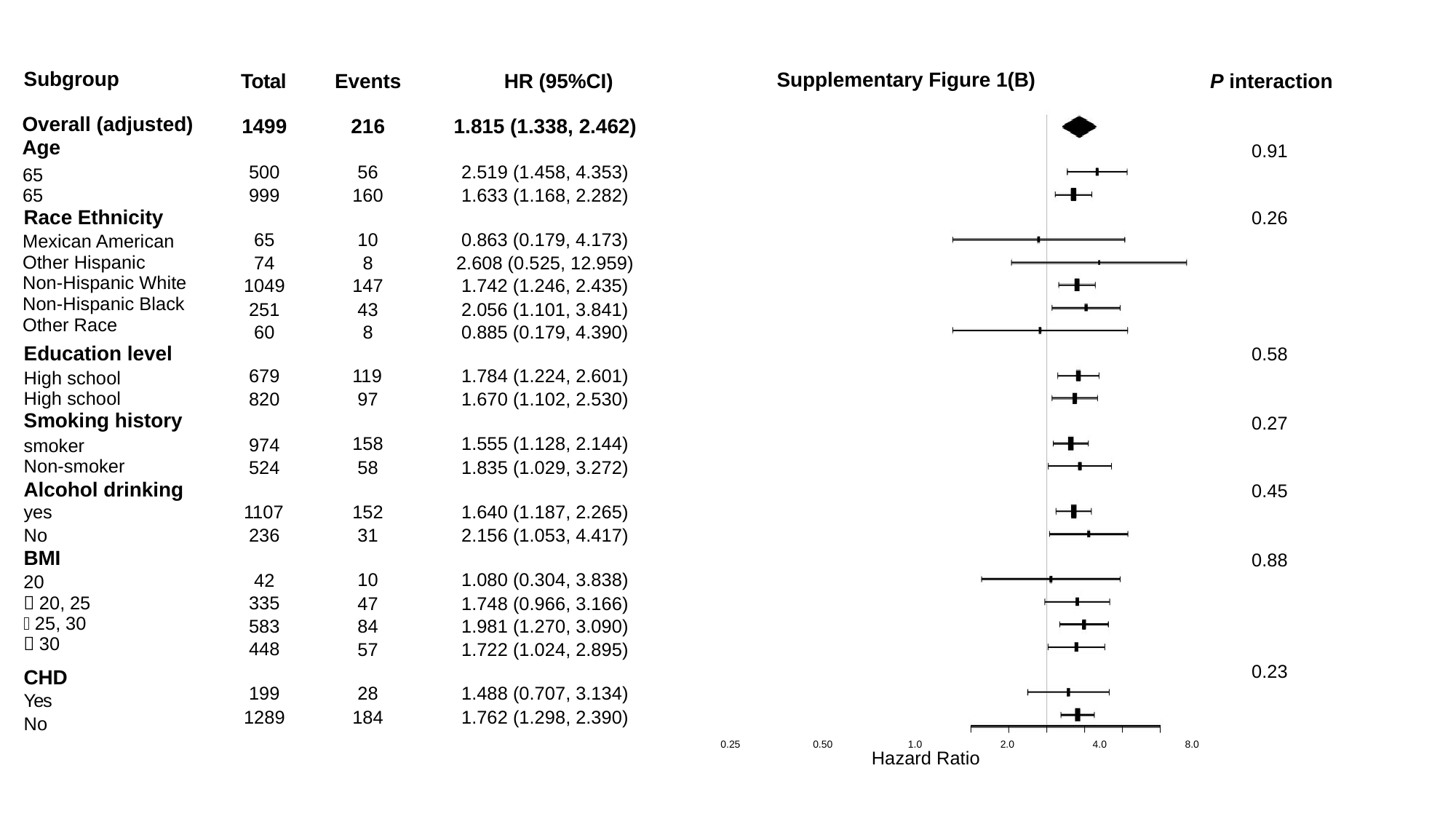

Supplementary Figure 1(B)
Subgroup
Total
1499
Events
216
HR (95%CI)
1.815 (1.338, 2.462)
P interaction
0.91
Overall (adjusted)
Age
500
999
56
160
2.519 (1.458, 4.353)
1.633 (1.168, 2.282)
Race Ethnicity
0.26
65
74
1049
251
60
10
8
147
43
8
0.863 (0.179, 4.173)
2.608 (0.525, 12.959)
1.742 (1.246, 2.435)
2.056 (1.101, 3.841)
0.885 (0.179, 4.390)
Mexican American
Other Hispanic
Non-Hispanic White
Non-Hispanic Black
Other Race
0.58
0.27
0.45
0.88
Education level
Smoking history
679
820
974
524
119
97
1.784 (1.224, 2.601)
1.670 (1.102, 2.530)
158
58
1.555 (1.128, 2.144)
1.835 (1.029, 3.272)
smoker
Non-smoker
Alcohol drinking
yes
No
BMI
1107
236
152
31
1.640 (1.187, 2.265)
2.156 (1.053, 4.417)
42
10
47
84
57
1.080 (0.304, 3.838)
1.748 (0.966, 3.166)
1.981 (1.270, 3.090)
1.722 (1.024, 2.895)
335
583
448
＞30
0.23
CHD
Yes
No
199
1289
28
184
1.488 (0.707, 3.134)
1.762 (1.298, 2.390)
0.25
0.50
1.0
2.0
4.0
8.0
Hazard Ratio

## Slide 4
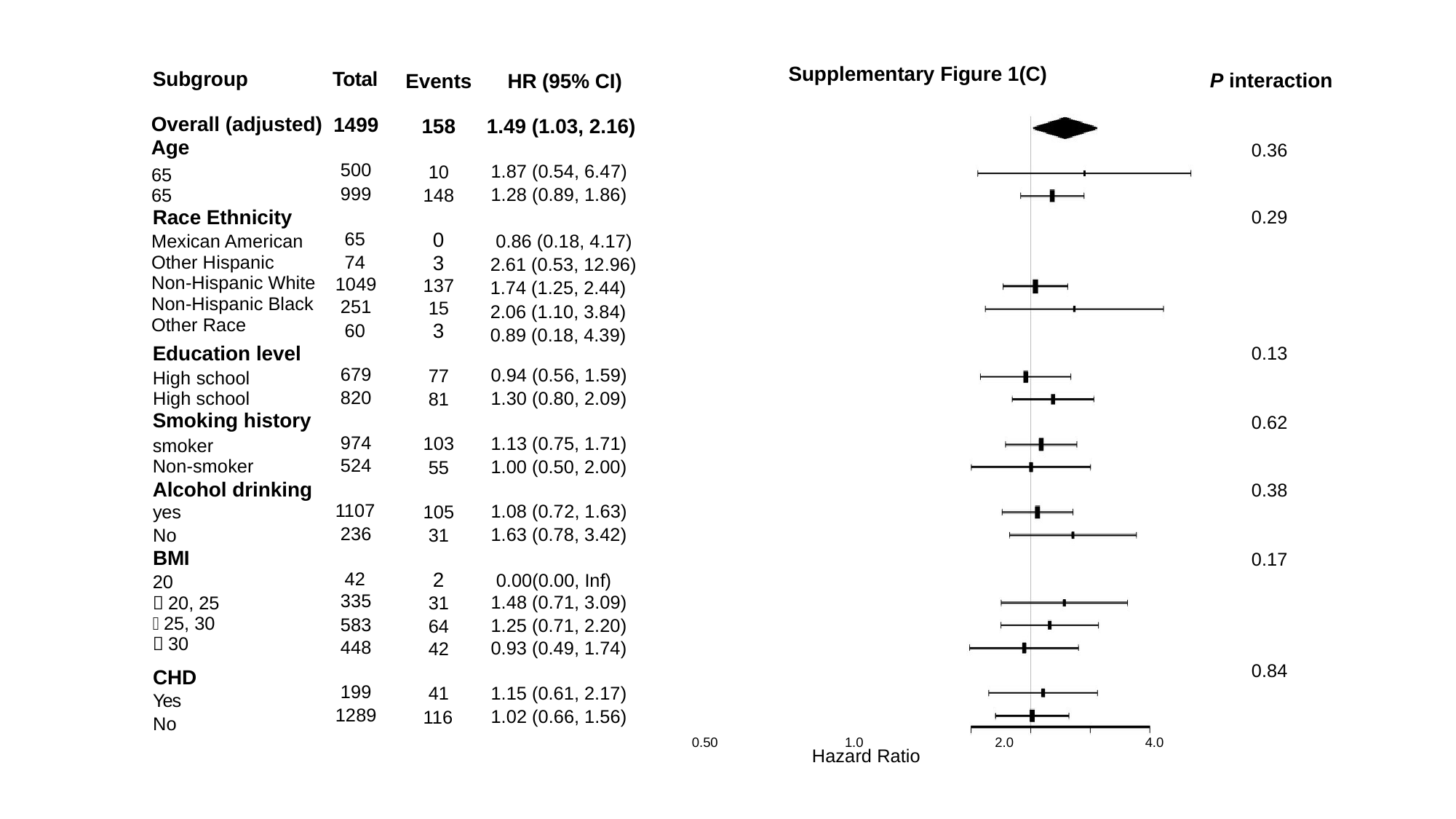

Supplementary Figure 1(C)
Subgroup
Total
1499
P interaction
0.36
Events
158
HR (95% CI)
1.49 (1.03, 2.16)
Overall (adjusted)
Age
500
999
1.87 (0.54, 6.47)
1.28 (0.89, 1.86)
10
148
0.29
Race Ethnicity
65
74
1049
251
60
0
3
137
15
3
0.86 (0.18, 4.17)
2.61 (0.53, 12.96)
1.74 (1.25, 2.44)
2.06 (1.10, 3.84)
0.89 (0.18, 4.39)
Mexican American
Other Hispanic
Non-Hispanic White
Non-Hispanic Black
Other Race
0.13
0.62
0.38
0.17
Education level
Smoking history
679
820
974
524
0.94 (0.56, 1.59)
1.30 (0.80, 2.09)
77
81
1.13 (0.75, 1.71)
1.00 (0.50, 2.00)
103
55
smoker
Non-smoker
Alcohol drinking
1107
236
1.08 (0.72, 1.63)
1.63 (0.78, 3.42)
yes
No
BMI
105
31
42
0.00(0.00, Inf)
1.48 (0.71, 3.09)
1.25 (0.71, 2.20)
0.93 (0.49, 1.74)
2
335
583
448
31
64
42
＞30
0.84
CHD
Yes
No
199
1289
1.15 (0.61, 2.17)
1.02 (0.66, 1.56)
41
116
0.50
1.0
2.0
4.0
Hazard Ratio
